# Impact of Calcium Propionate Supplementation on the Lactation Curve and Milk Metabolomic Analysis on Rambouillet Ewes

**DOI:** 10.3390/vetsci12020079

**Published:** 2025-01-22

**Authors:** Luis Fernando Pérez Segura, Hector A. Lee-Rangel, Rogelio Flores Ramirez, Juan Carlos García-López, Gregorio Álvarez-Fuentes, Anayeli Vázquez Valladolid, Pedro A. Hernández-García, Octavio Negrete Sanchez, Juan Antonio Rendon Huerta

**Affiliations:** 1 Instituto de Investigaciones en Zonas Desérticas, Facultad de Agronomía y Veterinaria, Centro de Biociencias, Universidad Autónoma de San Luis Potosí, San Luis Potosí 78321, Mexico; lpsyc95@hotmail.com (L.F.P.S.); jcgarcia@uaslp.mx (J.C.G.-L.); gregorio.alvarez@uaslp.mx (G.Á.-F.); anayeli.vazquez@uaslp.mx (A.V.V.); luis.negrete@uaslp.mx (O.N.S.); antonio.rendon@uaslp.mx (J.A.R.H.); 2 Coordinación para la Innovación y Aplicación de la Ciencia y la Tecnología (CIACYT), Universidad Autónoma de San Luis Potosí, Av. Sierra Leona núm. 550-2ª, Lomas de San Luis, San Luis Potosí 78210, Mexico; rogelio.flores@uaslp.mx; 3 Centro Universitario Amecameca, Universidad Autónoma del Estado de México, Amecameca 56900, Mexico; pedro_abel@yahoo.com

**Keywords:** ewes, gluconeogenic precursor, metabolomic analysis, milk compound, milk performance

## Abstract

In lactating ewes, energy demand increases for milk production, reserve mobilizations, and body weight maintenance. For reconversion to energy, ruminants require ruminal propionate production because it is the most predominant substrate for gluconeogenesis and one of the most relevant pathways since it allows an adequate supply of glucose. Calcium propionate supplementation is an alternative to increase glucose production by an external additive. Thus, the objective was to evaluate the effect of calcium propionate (CaPr) on milk production and milk metabolomic profile on lactating ewes. Sixteen Rambouillet (65.3 ± 6.2 kg BW; three years old) were randomly assigned one of two experimental treatments: (a) basal diet without supplementation (CP/0S) and (b) basal diet + 30 g d^−1^ of CaPr (CP/30S). The experimental period was from parturition day until day 60 (baby lamb weaning). A completely randomized design was used and analyzed with a mixed model. Initial and final lactating weight and milk production differed statistically (*p* < 0.05) between treatments. CP/30S led to differential changes (*p* < 0.05) in the lactation curve, showing significant milk production over eight-week measurements. Lactation peak (mL), maximum production (mL), and lactational persistency (d) were superior (*p* < 0.05) for supplemented ewes. An 11.4% variability was shown in a principal component analysis between treatments. For CP/0S, 63 bioactive compounds were detected, and 55 for CP/30S treatment. The metabolites detected in CP/0S showed that only fatty acid biosynthesis, biosynthesis of unsaturated fatty acids, and fatty acid elongation pathways were affected (*p* < 0.05) in milk. However, for CP/30S, metabolic pathways related (*p* < 0.05) were fatty acid biosynthesis, biosynthesis of unsaturated fatty acids, fatty acid elongation, phenylalanine metabolism, and steroid metabolism in milk samples. Calcium propionate supplementation increases milk performance and lactation persistency-induced changes in specific metabolic milk production pathways.

## Simple Summary

Lamb production in Mexico is focused on meat production. Some of the challenges faced are seasonality and production systems. The most common are extensive production systems in different regions of the country. Lamb production depends on survival, growth and successful weaning, and a fundamental factor is the milk and quality production. Energetic supplementation is a strategy to reach success in production systems. Calcium propionate is a gluconeogenic supplement that is used to produce glucose by ruminants. Regardless of the nutritive quality of milk commonly for protein, lactose, fat, and total solids, the milk provides numerous compounds that can give us information about the physiological processes that ewes suffer during their production; metabolomic analysis is a tool that can help us to understand the information about the relationship between compounds and metabolic processes. This paper study the effects of supplementing lactating ewes with calcium propionate and their participation in the milk metabolomic process.

## 1. Introduction

Mexico has an estimated sheep population of approximately 9 million [1], of which about 700,000 are Rambouillet breed. This breed was primarily utilized to obtain wool; however, due to a decline in production and price, the national flock has been constantly decreasing [2].

The nutritional status of the ewes during lactation significantly affects milk production and lamb development in various ways [3]. Some economic factors, such as diet quality, breed, season, and production system, can affect the nutrient status of the ewe during lactation [4]. Feeding management is an essential factor that affects the quantity and quality of milk produced. Maternal colostrum and milk yield of dam are essential for lambs to grow properly during the first weeks of their lives [5]. Milk serves as the first food in the mammal’s life, and its quality is crucial to ensure that the nutritional requirements of the young are met. Milk ewe composition could vary based on numerous factors, such as breed, health, age, lactation stage, and nutritional management [6]. A fundamental focus is to balance the relationship between production and product quality while addressing the metabolic aspects, which begin with feeding strategies.

During lactation, glucose requirements increase, stimulating fat mobilization to counteract energy deficit during the first third of lactation [7]. This energy deficit is associated with a negative energy balance (NEB), reduced milk production, and metabolic disturbances [8]. Propionic acid is the primary substrate for gluconeogenesis, a vital pathway that provides adequate glucose supply as recycled glucose is obtained from hepatic gluconeogenesis [9]. Propionate disposal establishes the amount of glucose that can be produced, which is correlated with gluconeogenesis hepatic function [10].

Gluconeogenic substrates offer an alternative to improve the glucose supply in ruminants. Calcium propionate supplementation is one such option, delivering propionic acid to an external source rather than relying on starch fermented in the rumen; this can be used to modulate the energy that animals feed on during the productive phases [11]. Perez-Segura et al. [12] reported that supplemented ewes with CaPr in different thirds of gestation could be a solution to improve productivity performance and health status due to fact that the molecule in the rumen can partition in propionate, and calcium gave beneficial effects to ewes.

Lactating ruminants require a substantial amount of glucose as a substrate for synthesizing lactose, an osmoregulator of water, into the mammary gland, resulting in major milk production [13]. Increasing gluconeogenic substrates in the liver thus serves as a method to increase milk production [14]. Nevertheless, not only was the production volume affected, but milk quality can also be influenced by gluconeogenic use in ewes’ diet. Ahmadzadeh et al. [15] reported that using propylene glycol as a gluconeogenic substrate in ewes during late pregnancy and parturition reduced milk fat. These are possibly due to alterations in the fat rumina precursors as acetate and butyrate.

Currently, metabolomics as a tool provides us with an analysis of the end products of complex genetic, epigenetic, and environmental interactions, which represents a valuable resource to deepen and improve our understanding of physiological processes affecting livestock production [16,17]. Over the years, there has been increasing interest in using metabolic markers to identify more efficient animals. Thus, we hypothesized that calcium propionate supplementation in lactating ewes could enhance lactating performance and alter milk composition, affecting functional pathways. Therefore, this study aimed to explore the effect of supplementation of calcium propionate on milk production, weight changes, and milk metabolic profiles in lactating ewes.

## 2. Materials and Methods

### 2.1. Ethics

Before initiating the study, all animal procedures were reviewed and approved by the Committee for the Ethical Use of Animals in Research of the Universidad Autónoma de San Luis Potosi. This study adhered to the regulations and norms established in the Official Daily of the Federation, Mexico, following federal law concerning animal care, farms, reproduction, production, and breeding centers, and in accordance with the principles of animal welfare [18].

The experiment was conducted at the ovine unit of the animal science experimental station of the Faculty of Agronomy and Veterinary of the Autonomous University of San Luis Potosi, Soledad de Graciano Sanchez, San Luis Potosi, Mexico (Latitude 22°14′0.58″; Longitude 100°50′48.5″).

### 2.2. Animals and Diets

The study involved 16 multiparous lactating Rambouillet ewes (65.3 ± 6.2 kg BW; 3 years old) with an average prolificacy of 1.3 baby lambs/ewe. These animals were randomly assigned one of two experimental treatments: (a) basal diet (Table 1) without supplementation (CP/0S) (*n* = 8) and (b) basal diet supplemented with 30 g/day of CaPr (CP/30S) (*n* = 8). The experimental period lasted from parturition day to weaning (60 days); these ewes were housed in group pens (*n* = 4 per pen) equipped with feeders and drinkers. The ewes were fed with alfalfa hay and corn silage and 300 g/ewe/d of commercial concentrate (Vali Commercial^®^, San Luis Potosi, Mexico). The manufacturer reported that the chemical diet contains 15.9% of crude protein, 3.0% of ether extract, 12% of crude fiber, 7.5% of ash, and 90% of dry matter. In particular, feed was offered ad libitum to ensure 10% of refusals. Feed was given at 8:00 and 15:00 h, with the experimental diets provided throughout the 60-day period. Recent studies have demonstrated that this level of CaPr can produce productive and metabolic changes in finishing lambs [19]. Calcium propionate was top-dressed on the feed provided to the ewes. The diet samples were collected weekly for further analysis for determination of dry matter (DM) and crude protein (CP) according to AOAC [20]. Neutral detergent fiber (with heat stable amylase) and acid detergent fiber were determined according to Van Soest et al. [21].

### 2.3. Sample Collection and Calculations

After parturition, ewes were milked every 10 days until day 60. Milk production was measured every 10 days, as described by Reynolds et al. [22]. Ewes were separated from their young at 08:00 h and immediately milked by hand. This milk collected during this initial milking would be offered to the lambs. After 3 h, an injection of oxytocin (20 IU) was administered intramuscularly. Ten minutes post-injection, the ewes were milked and milk production was recorded. Milk samples were frozen until further analysis.

The lactation curve was estimated using the incomplete gamma function or Wood’s model [23] because its three parameters have a relationship with the biology of the lactation curve [24]. In addition, this model shows a good fit in studies of milk production in ewes [25,26]. The model is represented as *yt* = *at^b^ e*^−*ct*^, where *yt* is the milk production in the time period, *e* is the base for the natural logarithm, and *a*, *b*, and *c* are the parameters of the curve. a represents the milk production at the beginning of lactation, while *b* and *c* represent the limit in the decline of milk production at the beginning.

### 2.4. Analytical Methods

Milk samples for each ewe were collected in conical tubes of 10 mL during three out of the five milkings: one initial, one intermediate, and one final, obtaining 24 samples by treatment for later analysis. These samples were stored at −20 °C until further analysis. Milk compounds were extracted using an ultrasonic processor (GEX130, 115 V 50/60 Hz). A 75/25% mix of hexane and acetone was added to 1 mL of milk to make the extraction. Subsequently, the organic phase was separated, concentrated into 1 mL extracted mixture, and evaporated (Zymark, Turbovap LV Concentration Evapotarot, NB, Clackamas, OR, USA) for final analysis. Milk characterization was performed using gas chromatography (GC-HP 6890) coupled to mass spectrophotometry (MSHP 5973). This system was equipped with a capillary column of 60 m length, 0.255 mm diameter, and 0.25 µm film thickness (HP 5 MS, Agilent). The temperature program began at 70 °C for 2 min, which was increased to 250 °C at a rate of 20 °C/min, to 290 °C at a rate of 5 °C/min, to 300 °C at a rate of 1 °C/min, and then to 310 °C at a rate of 5 °C/min, where it was held for 36 min. The injector temperature was set at 250 °C in splitless mode, with a helium flow rate of 1 mL/min. Mass spectrophotometry was programmed to identify compounds in SCAN mode (50–500 *m*/*z*).

### 2.5. Statical Analysis

The data were analyzed using a completely randomized design (CRD) with two dietary treatments and eight replicates per treatment. A General Linear Model (GLM) was employed to evaluate the effects of dietary treatments on response variables such as volatile fatty acid concentrations and microbial composition. The mathematical model used was as follows:

Y_ij_ = μ + T_i_ + ε_ij_

where Y_ij_ = observed response variable for the j-th replicate within the i-th treatment; μ = overall mean; T_i_ = fixed effect of the i-th treatment (i = 1, 2, 3, 4); and ε_ij_ = random error associated with the j-th replicate within the i-th treatment, assumed to be normally distributed (ε_ij_~N(0, σ^2^)). Tukey’s Honest Significant Difference (HSD) test was performed for post hoc multiple comparisons at a significance level of α = 0.05 to compare treatment means. Statistical analyses were conducted using SAS software (version 9.0).

The MetaboAnalyst 6.0 tool was used to perform a multivariate statistical analysis. Initially, to normalize the data, a log transformation was used to correct the heteroskedasticity and decrease the mask effects. Partial Least Square Discriminant Analysis was performed for all metabolites obtained; after that, a Variable Importance Projection was obtained to rank the metabolites according to their importance in discriminating groups for the pathways of different metabolites in each comparison according to the KEGG database (https://www.metaboanalyst.ca/MetaboAnalyst/ModuleView.xhtml. accessed on 25 June 2024).

## 3. Results

### 3.1. Performance of Ewes

There were statistically significant differences for the initial lactating weight (*p* ≤ 0.04), final lactating weight (*p* ≤ 0.03), and milk production (*p* ≤ 0.03). However, there were no statistically significant differences (*p* ≤ 0.06) among the groups for ewe weight difference (Table 2).

**Figure 1:**
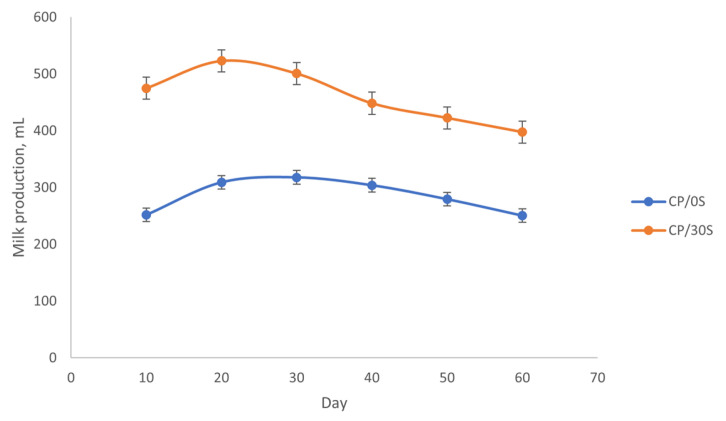
Lactation curves for daily milk production in ewes supplemented with 30 g/day of calcium propionate (CP/30S) and nonsupplemented ewes (CP/0S) over 60 days from the parturition.

Figure 1 shows the lactation curves adjusted over 60 days postpartum for CP/0S and CP/30S. Calcium propionate significantly (*p* ≤ 0.05) improved milk production of ewes supplemented compared to nonsupplemented ewes.

The estimated performance parameters for adjusted curves were as follows: a = 88.76, b = 0.06, and c = 0.02. The production peak was present on day 24 with a maximum production of 344.63 mL of milk and a lactation persistency of 12 days for control treatment. For ewes supplemented, the parameters were significantly (*p* ≤ 0.05) higher, with a = 517.34, b = 0.03, and c = 0.06. The production peak occurred on day 10 with a maximum production of 521 mL of milk and a lactation persistency of 22 days (Table 3).

### 3.2. Milk Metabolomic Profile

**Figure 2:**
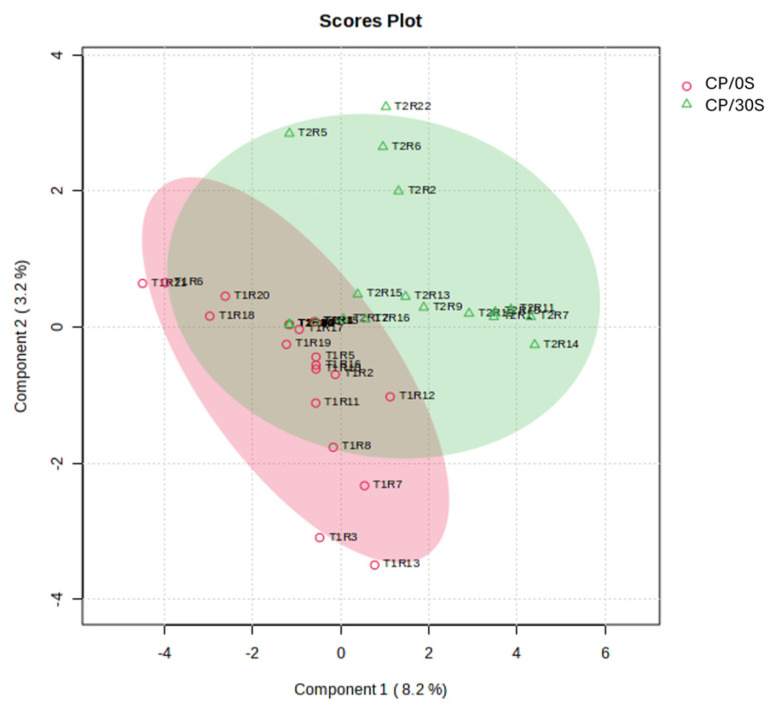
Partial least squares discriminant analysis (PLS-DA) loading map for volatile CP/0S and CP/30S.

With the information obtained from the analysis of the identified metabolites in milk, a partial least square of discriminant analysis (PLS-DA) was conducted to interpret the difference between treatments. A clustering between CP/0S and CP/30S treatments was revealed (Figure 2), with a maximum variation of 8.2% among the database treatments.

**Figure 3:**
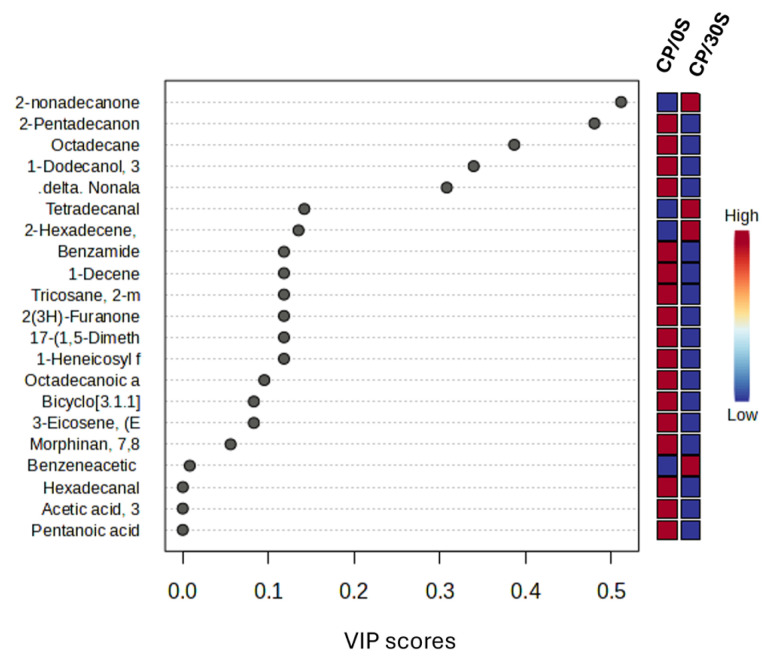
Variable importance in projection test for CP/S0 and CP/30S ewe milk compounds.

The variable importance projection for both treatments shows that metabolites found in ewe milk are more related to CP/0S. According to loadings, only 2-nonadecanone, tetradecane, 2-hexadecane, and benzeneacetic present a variable importance in projection score with a high relation to CP/30S (Figure 3).

**Figure 4:**
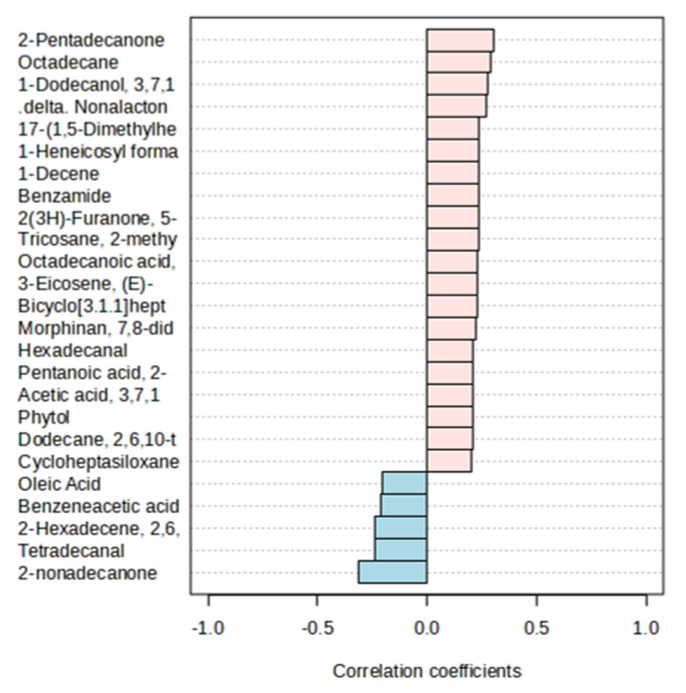
Spearman’s rank correlation coefficient reports the correlation of 25 most important compounds between treatments.

In Figure 4, the correlation coefficients show that twenty biocompounds found in ewe milk present a positive correlation with both treatments; five were negatively correlated (oleic acid, benzene acetic acid, 2-hexadecene, tetradecane, and 2-nonadecanone).

**Figure 5:**
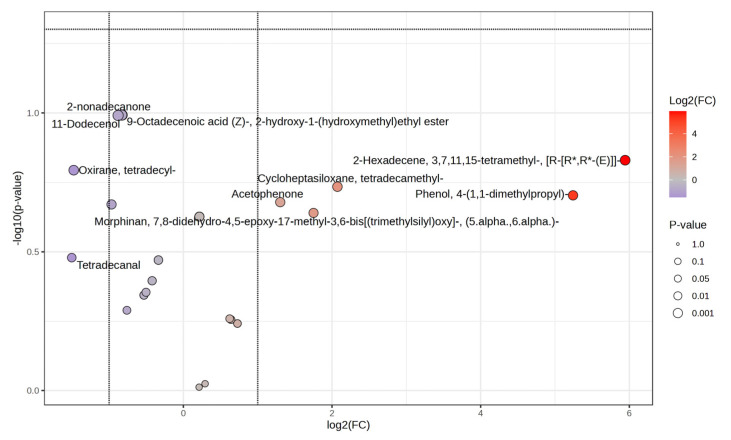
Volcano plot of ewe milk biocompound analysis from CP/S0 and CP/S30 treatments; red dots are upregulated, and purple dots are downregulated metabolites identified.

The compounds detected in milk analyzed from ewes in treatments were 63 and 55 compounds for CP/0S and CP/30S, respectively. Of compounds detected in both treatments, five (tetradecanal, morphinan, oxirane, dodecenal and 2-nonadecanone) are downregulated (fold change ≤ 1, *p* < 0.05; Figure 5), whereas another five compounds (acetophene, cycloheptasiloxane, dimethypropil, and hexadecane) are upregulated (fold change ≤ 1, *p* < 0.05; Figure 5).

**Figure 6:**
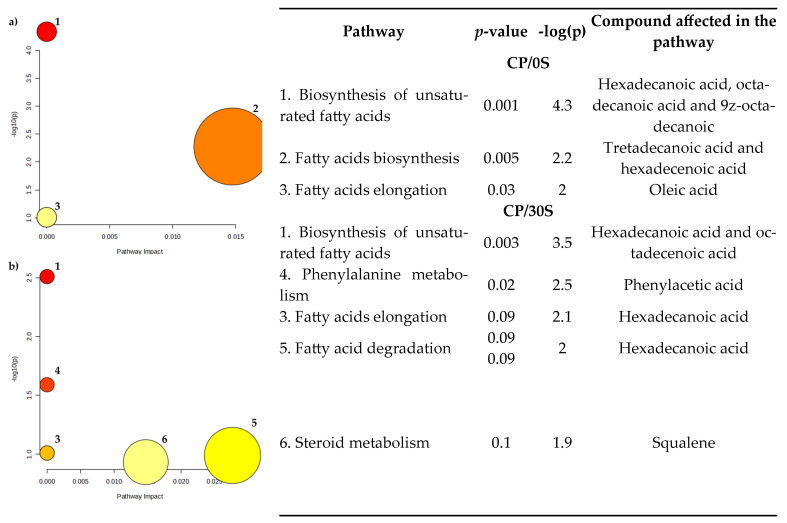
Pathway topology (functional analysis) according to KEEG pathway folder; identification of differential metabolites in ewe milk supplemented with calcium propionate.

The pathways identified according to the KEGG database indicated that unsaturated fatty acid biosynthesis, fatty acid biosynthesis, and fatty acid elongation were significantly affected (*p* < 0.05) in both treatments. Notably, the phenylalanine and steroid metabolism was also affected (*p* < 0.05) by calcium propionate supplementation (Figure 6).

## 4. Discussion

Table 2 indicates that lactating ewes supplemented with CaPr showed alterations in milk production and body weight changes. Mendoza et al. [27] and Cifuentes-Lopez et al. [19] reported that the CaPr can provide a certain amount of metabolic energy as propionic acid apport, calculated at 3.96 Mcal/kg of DM. This energy contributes directly to the metabolism of ewes to maintain body weight and increase milk production. Similarly, Hashem and El-Zarkouny [28] found that a gluconeogenic precursor (propylene glycol) used in lactating ewes reduces weight loss. These gluconeogenic pre-cursors supply more energy to maintain body weight deposit. Additionally, the use of CaPr in finishing lambs increases the insulin linearly with the CaPr concentration in the diet (30, 35, and 40 g/lamb/day). However, this increase is not related to glucose plasma concentration [19]. In lactating animals, peripheral tissues exhibit a significantly reduced response to insulin during early lactation, but this response improves as lactation advances [29]. In contrast to our findings, the milk production reported by Hashem and El-Zarkouny [28] was unaffected by CaPr supplementation, glucose uptake by the mammary gland is minimally influenced by insulin, and the gland does not exhibit substantial insulin-dependent glucose transport [30]. Consequently, these physiological processes may shift the balance between peripheral tissues and the mammary gland in favor of the peripheral tissues, promoting increased body weight rather than milk production.

Studies report using plasma as the matrix of choice for metabolomic analysis because it detects a more significant load of differential metabolites; however, other studies highlight milk samples as a practical and valuable option as they are easily accessible during regular milk recording on farms [28]. Moreover, in various scenarios, matrices may be more closely related to different physiological processes than others (e.g., milk to mammary metabolism and plasma to rumen fermentation, digestion, and liver metabolism) [29]. The profile analyzed was interesting because the primary compound detected corresponded to aroma and flavor products. This result allows us to obtain information about the final consumer’s perception of the off-flavor attribute of the milk or products obtained [30]. Sanchez et al. [31] mentioned that the use of analytical methods to establish chemical fingerprints commonly generates a big database where many classes of compounds are found, such as alcohols, aliphatic hydrocarbons, aromatic compounds, ketones, aldehydes, and free fatty acids. The results of Spearman’s correlation showed that most of the compounds detected belong to the classification of alkanes (octadecane, 1-decene, tricosane, and eicosane), fatty acids (oleic acid, octadecanoic acid, heneicosyl, hexadecanoic, 9z-octadecanoic) ketones (2-pentadecanone), aromatics (benzamine, furanone, bicyclo heptane, pentanoic acid, phytol), amides (di-methyl hexanal), aldehydes (hexadecanal), and aliphatic amines (dimethyl hexanal) that are related to both treatments. These compounds have been reported in fresh goat milk with the quantity or specific profile decreased or increased depending on time and condition of storage [32,33].

According to our findings, fatty acid metabolism is the primary metabolic pathway affected. Fatty acids in milk could originate from plasma lipoproteins or be synthesized de novo in the mammary gland [34,35,36]. During early lactation, the NEB is considered a normal condition where the body fat reserves are extensively mobilized in non-esterified acids [35,36,37]. In this study, the biosynthesis of unsaturated fatty acids (BUFAs) in milk was explicitly affected by hexadecanoic (palmitic acid) and octadecanoic (stearic acid) acids for both treatments and by 9z-octadecanoic acid (linoleic acid) in CP/0S. A relation between unsaturated fatty acids (UFAs) and saturated fatty acids (SFAs where UFAs are in a higher concentration with respect to SFAs is indicative of reduced de novo synthesis activity; this depends on a tricarboxylic cycle that takes place in mitochondria [36,37,38,39]. Our results show that the BUFA pathway has a higher impact on CP/0S than CP30S. In agreement with this, Roy et al. [38,40] reported that lower rates of circulant propionic acid may result in the most liver activity for oxidative metabolism as a result of the increase in the tricarboxylic acid cycle. Thus, we assume that the CP/30S could reduce hepatic oxidation, resulting in a reduction in plasmatic beta-hydroxybutyrate concentration, resulting in the prevention of possible ketogenesis as a result of the increase in insulin secretions that reduce the lipolysis of adipose tissue. Notably, the presence of hexadecanoic, octadecanoic, and 9z-octadecanoic acids in milk fat can be a predictor of subclinical ketosis. The 9z-octadecanoic acid (CP/0S) was found in hyperketonemic ewes, implying that high concentrations of this fatty acid can be used as an early biomarker for subclinical ketosis in ewes [39,41]. The results indicate that nontargeted metabolomics offers valuable information on the metabolic pathways affected by energy-based supplementation in lactating ewes. This represents a viable option for identifying changes in the animal physiological processes and even establishing metabolomic biomarkers in the animal [31,32]. This idea supports the hypothesis that using CaPr as a supplement in lactating ewes can prevent metabolic disorders with deficient nutrition, as could be the case with ewes in our control group, because this compound was found there. In this way, for the formation of UFA, not only is de novo synthesis used, but also, the activity of Δ5 desaturase acts to incorporate some fatty acids such as tetradecanoic acid (myristic acid) rather than desaturating some other saturated fatty acids [40]; this implies that in our findings, the fatty acid biosynthesis pathway can be affected in this way on the CP/0S.

Fatty acid elongation was another pathway affected; this could be explained because propionate indirectly contributes to branched-chain fatty acid synthesis through methylmalonyl-CoA incorporation. Early integration of methylmalonyl-CoA during chain elongation produces anteiso-fatty acids, while later integration yields branched-chain fatty acids with a methyl group near the carboxyl end [41]. These fatty acids are present in sheep and goat adipose tissue and milk fat. They are still absent in cow’s milk, reflecting low methylmalonyl-CoA incorporation in bovine mammary tissue [42,43]. These observations highlight species-specific differences in fatty acid synthesis between cattle and small ruminants.

In contrast, steroid metabolism was affected by CaPr supplementation. Payne and Hales [44] noted that steroidogenesis refers to the biosynthesis of steroid hormones derived from cholesterol. However, squalene is the compound that serves as an intermediary for the formation of cholesterol. Our results of CP/S30 treatment suggest that squalene is the compound involved in the steroid pathway. Propionate as a supplement is linked to carbohydrate metabolism and inhibits cholesterol synthesis. If CaPr alters the ruminal butyrate concentration, it can be converted into β-hydroxybutyric acid, which plays a role in the metabolic activities of tissues like the liver and muscles, supplying the energy required by the body [45,46].

Not only were fatty acids affected in the milk metabolome, but also, aroma components such as phenylacetic acid were found in milk from CP/30S, related to the phenylalanine pathway. Phenylacetic acid is a product of phenylalanine metabolism derived by the Strecker-type reaction, which involves the oxidation of phenylalanine derived from a thermal process. It could also originate as a metabolite of the amino acid tryptophan or from animal feeds like aromatic herbages [47]. There is limited information about phenylacetic acid. Phenylalanine is an aromatic amino acid involved in protein synthesis. Lamichhane et al. [48] mentioned that these pathways result in diverse metabolites that mediate nervous signaling to eliminate reactive oxygen species in the brain and maintain animal health. Moreover, as a part of phenylalanine metabolism, this can be converted to nicotinamide, and the result is significant energy generation through diverse hormone regulation and glucose metabolism [49], which could be related to our results for an increase in milk lactation performance in CP/30S. Tong et al. [50] propose that changes in amino acid metabolism could partially explain differences in milk yield and quality.

## 5. Conclusions

Supplementing lactating ewes with 30 g/day of calcium propionate 60 days after birth increases milk production and reduces body weight loss. The metabolomic analysis of milk presents some compounds such as hexadecenoic, octadecanoic, 9z-octadecanoic, tetradecanoic, oleic, and phenylacetic acids, as well as squalene, which affects the fatty acid biosynthesis, fatty acid biosynthesis, fatty acid elongation, and phenylalanine and steroid metabolism pathways in milk metabolome analysis. These results indicate that calcium propionate could be used as a gluconeogenic supplement in lactating ewes to improve milk performance and influence the metabolic pathways associated with milk production.

## Figures and Tables

**Table 1:** Basal diet and chemical composition fed to the lactating ewes.

Ingredients	g kg^−1^ of DM
Corn silage	500
Alfalfa hay	500
**Chemical composition, g kg^−1^ of DM**	
Dry matter	646.65
Crude Protein	157
Neutral Digestion Fiber	470
Acid Digestion Fiber	276.5
Ether extract	31.45
Ash	92.1

**Table 2:** Effects of supplementation over 60 days with CaPr on performance production in lactating Rambuillet ewes.

Item	CP/0S	CP/30S	*p*-Value	SEM
Initial lactating ewe weight, kg	67.8 ^a^	62.3 ^b^	0.04	7.2
Final lactating weight, kg	64.7 ^a^	60.5 ^b^	0.03	5.8
Difference, kg	3.1 ^a^	1.8 ^b^	0.06	0.99
Milk production, mL	315.6 ^b^	472.7 ^a^	0.04	36.1

CP/0S: nonsupplemented ewes; CP/30S: 30 g day-1 of calcium propionate-supplemented ewes; SEM, standard error of the mean; ^a,b^ means within a different letter differ statically (*p* < 0.05).

**Table 3:** Lactation performance (lactation peak, maximum production, and lactation persistence) for ewes supplemented and nonsupplemented with calcium propionate over 60 days of lactation.

Item	CP/0S	CP/30S	*p*-Value	SEM
Lactation peak, day	24 ^a^	10 ^b^	0.01	1.73
Maximum production, mL	344 ^b^	521 ^a^	0.01	12.5
Lactation persistence, day	12 ^b^	22 ^a^	0.02	0.62

CP/0S: nonsupplemented ewes; CP/30S: 30 g day^−1^ of calcium propionate-supplemented ewes; SEM, standard error of the mean; ^a,b^ means within a different letter differ statically (*p* < 0.05).

## Data Availability

Data are contained within the article.
